# Fatal Postoperative *Candida glabrata* Septicemia in a Child with Congenital Heart Disease

**DOI:** 10.4103/0974-777X.52986

**Published:** 2009

**Authors:** Vasant Baradkar, Shripad Taklikar, Simit Kumar

**Affiliations:** *Department of Microbiology, Lokmanya Tilak Municipal Medical College & General Hospital, Sion, Mumbai - 400 002, India*

**Keywords:** *Candida glabrata*, Septicemia, Congenital heart disease

## Abstract

The incidence of candidemia has been reported to be high in some cardiovascular surgery units. Congenital heart disease has been considered a risk factor for acquisition of candidemia. This present case is a postoperative *Candida glabrata* in a child with congenital heart disease. A 3-year-old child, a previously diagnosed case of situs-solitus D loop situs with double outlet right ventricle, ventricular septal defect, pulmonary stenosis and large ostium secundum atrial septal defect, was admitted with history of effort intolerance. A left modified Blalock-Taussig shunt was performed and then the child underwent closure of the ventricular septal defect and the atrial septal defect. On the third day the patient developed fever. *Klebsiella pneumoniae* was isolated from blood which responded to piperacillin + tazobactam but on the twelfth day, the patient again developed fever spikes. The blood cultures performed at this time showed repeated isolation of *Candida glabrata.* Amphotericin B was started but still the patient deteriorated and died on the 22^nd^ day after operation. The antifungal susceptibility of the isolate performed showed that the isolate was resistant to Amphotericin B.

## INTRODUCTION

Nosocomial candidemia has increased during the past two decades and now accounts for 10-20% of all nosocomial bloodstream infections. It is a serious infection with estimated case-fatality rates of 20% for children and 50% for adults. The National Nosocomial Infections Surveillance System reported that *Candida species* has become the fourth most common nosocomial blood culture isolate in the United States.[1]

Candidemia has also become an important problem in the pediatric population, indeed neonatal intensive care units (ICUs) are the sites of most candidemia outbreaks. The incidence of candidemia has been reported to be high in some cardiovascular surgery units.[2]

Numerous risk factors have been identified for candidemia, including use of total parenteral nutrition (TPN), broad spectrum antibiotics, presence of an indwelling vascular catheter, immunosuppressive therapy, hospitalization in an intensive care unit, colonization with *Candida species*, extensive burns and treatment with corticosteroids.[1] Congenital heart disease (CHD) has also been considered a risk factor for acquisition of candidemia.[2] Here we report a fatal case of postoperative *Candida glabrata* septicemia in a child with congenital heart disease, who later succumbed in spite of receiving Amphotericin B treatment, as the isolate was later found to be resistant to Amphotericin B by the API system.

## CASE REPORT

The case reported herein is a fatal case of *Candida glabrata* septicemia in a child with congenital heart disease. The 3-year-old male child was admitted with a history of effort intolerance. The patient was diagnosed as a case of congenital cyanotic heart disease two years back. There was no significant history of any disease in the family. Two dimensional echocardiography and color Doppler revealed situs-solitus D loop situs (SDS) with double outlet right ventricle (DORV), ventricular septal defect (VSD), pulmonary stenosis (PS) and large ostium secundum atrial septal defect. A left modified Blalock–Taussig shunt was performed for the cyanotic spells. The patient had an uneventful early postoperative course. The patient was admitted for elective total intracardiac repair. On the third day of admission, the child underwent closure of VSD and ASD, right ventricle – pulmonary artery conduit. The patient developed a complete heart block and required inotropes for seven days. The patient was maintained on a temporary pacemaker. The patient was on a prophylactic cover of amikacin and cefotaxime. The immediate postoperative period was uneventful.

On the fifth day the patient developed fever, for which routine blood investigations were sent in along with blood cultures. Repeated blood cultures collected from different body sites showed growth of *Klebsiella pneumoniae*, which was sensitive to piperacillin + tazobactam. The patient was then started with amoxycillin + clavulanic acid and piperacillin + tazobactam to which the patient responded well with the subsidence of fever.

However, meanwhile the child developed oral candidiasis. On the 12^th^ postoperative day the patient again had spikes of fever. The blood culture performed at this time showed repeated isolation of yeast, which was further identified to be *Candida glabrata* by the growth observed on cornmeal agar (CMA). The isolate was confirmed by repeated isolation and sugar assimilation tests. Based on the blood culture reports the patient was started on intravenous Amphotericin B. In spite of the therapy the patient succumbed on the 22^nd^ postoperative day, despite having received three days of amphotericin B, during which the blood cultures continued to remain positive for candida and no other bacterial pathogen was isolated.

The antifungal susceptibility testing was performed by the API system. The reports received after one week showed that the isolate was resistant to Amphotericin B and azoles and susceptible to caspofungin, but the patient had already succumbed before he could be started on caspofungin therapy.

## DISCUSSION

Candidemia is defined of *Candida species* from a blood culture in the presence of clinical features of infection. The association has been found between *Candida* colonization and candidemia.[1] *Candida species* were frequently isolated from different clinical samples, obtained for any reason from patients who later had candidemia, thus suggesting that colonization probably precedes infection, as previously reported by Vosset *et al.*,[3] Bonter *et al*.[4] and Martino *et al*.[5] The child in this case had oral candidiasis followed by dissemination to blood. The conclusion that the cause of death was candidemia was drawn from the fact that before the patient developed candidemia, his blood cultures revealed growth of *Klebsiella pneumoniae*, for which he was treated successfully with antibiotics with the improvement in the clinical status, with a five day apyrexial interval, followed by the development of oral candidiasis and the worsening in the patient's clinical status, with the blood cultures showing features of candidemia and no other bacterial pathogen being isolated from the blood cultures. The cultures from the peripheral lines, central line tip and urinary catheters also did not show any growth of candida, suggesting that the candidemia could be due to the colonization of the gastrointestinal tract as evidenced by the oral thrush followed by dissemination into the blood.

Historically *Candida glabrata* has been considered to be a relatively nonpathogenic saprophyte of normal healthy individuals rarely causing infection. However, following widespread and increased use of immunosuppressive therapy and broad-spectrum antibiotic therapy, the frequency of mucosal and systemic infections caused by *Candida glabrata* has increased significantly. [1–5]

Though *Candida glabrata* has emerged as an important nosocomial pathogen, infection by this species is associated with high mortality rates. *Candida glabrata* is of special importance because of its innately increased resistance to antifungal drugs, especially azoles. The reason behind the emergence of this species as predominant pathogen could be selection of lesser susceptible species due to frequent use of Fluconazole as prophylaxis. Most reports describing the epidemiology are retrospective, while a few studies have evaluated independent risk factors associated with nosocomial *Candida glabrata* acquisition and subsequent infection. [1–6] A recent multivariate prospective case control analysis along with molecular analysis of *Candida glabrata* demonstrated that a patient with new acquisition of *Candida glabrata* had a longer duration of hospitalization.[6]

Previous understanding of the pathogenesis of *Candida glabrata* colonization and infection assumed that organisms responsible for disease were endogenously acquired.[6] A similar finding was observed in this case report.

*Candida glabrata* and other *candida species* may cause fungemia in hospitalized patients. The risk factors for this include preceding surgery, administration of broad- spectrum antibiotics, patients on chemotherapy and steroids. In the present case the patient had undergone a major surgery, and was on broad-spectrum antibiotics, before the development of oral candidiasis, which probably acted as a source for the fungemia.

To conclude, in a hospitalized patient who has undergone major surgery and has been on broad-spectrum antibiotics, if the patient's pyrexia does not subside or reappears after a prolonged period of the patient being on broad-spectrum antibiotics, a differential diagnosis of fungemia should be considered by the physician. On the part of the laboratory, early speciation of especially the non-albicans *candida species* and if possible performing the antimicrobial susceptibility could help reduce the morbidity and mortality associated with such cases. The antimicrobial susceptibility pattern is especially more important in the cases of the incriminating agent being *Candida glabrata*, as it has been reported earlier to be resistant to Amphotericin B, with caspofungin being the treatment of choice. [1–6]
